# Energy Structure of Repeated On-Ice Efforts and Its Dependence on the Aerobic Capacity of a Hockey Player

**DOI:** 10.3390/sports14030116

**Published:** 2026-03-17

**Authors:** Tomasz Gabrys, Radoslaw Chruscinski, Anna Pilis, Arkadiusz Stanula, Kazimierz Mikolajec

**Affiliations:** 1 Faculty of Health Science, University of Applied Science, 48-300 Nysa, Poland; tomaszek1960@o2.pl; 2 Sport Centrum Faculty of Education, University of West Bohemia, 301 00 Pilsen, Czech Republic; 3 Silesian Ice Hockey Federation, 40-431 Katowice, Poland; 4 Department of Medical Science and Physiotherapy, Collegium Medicum, Jan Dlugosz University, 42-200 Czestochowa, Poland; a.pilis@ujd.edu.pl; 5 Laboratory of Sport Performance Analysis, Institute of Sport Sciences, Jerzy Kukuczka Academy of Physical Education, 40-065 Katowice, Poland; a.stanula@awf.katowice.pl; 6 Department of Team Sports Games, Jerzy Kukuczka Academy of Physical Education, 40-065 Katowice, Poland; k.mikolajec@awf.katowice.pl

**Keywords:** ice hockey, repeated high-intensity effort, energy system contribution, aerobic capacity

## Abstract

Background: Ice hockey is characterized by repeated short-duration, high-intensity efforts interspersed with brief recovery periods, requiring a complex interaction of aerobic and anaerobic energy systems. The aim of this study was to determine the energetic structure of repeated high-intensity on-ice sprint exercise in ice hockey players by quantifying the relative contributions of the oxidative, glycolytic and ATP–PCr energy systems. Methods: 14 male semi-professional ice hockey players performed the 30–15_IIT_ followed by the Repeated High-Intensity Effort (RHIE) on-ice. Oxygen uptake was measured breath-by-breath, blood lactate concentration and energy system contributions were estimated using a three-component PCr–La–O_2_ model. Results: The RHIE on-ice was characterized by a dominant aerobic contribution (63.1 ± 2.6%), followed by phosphagen metabolism (29.8 ± 2.9%), with a relatively small glycolytic contribution (7.4 ± 1.5%). Conclusions: No significant relationships were observed between maximal oxygen uptake (VO_2max_) and the RHIE performance parameters, energy system contributions or lactate responses, except for a moderate relationship between absolute VO_2max_ and absolute aerobic work. In contrast, parameters determined at the anaerobic threshold showed more consistent relationships with absolute metabolic work. These findings indicate that repeated high-intensity on-ice performance in ice hockey is largely independent of VO_2max_ and is more closely related to individual energetic profiles and metabolic tolerance.

## 1. Introduction

Ice hockey represents a team sport, characterized by high-intensity, intermittent exercise demands. It is estimated that nearly 50% of the total distance covered by players is performed at high skating velocities, accompanied by numerous stops, starts, rapid accelerations, and braking phases [1,2,3]. This type of activity is associated with a substantial physiological load with a pronounced contribution of anaerobic processes particularly glycolytic metabolism [4]. As demonstrated by Lignell et al. [3], these efforts typically last 3–5 s, occurring 4–10 times per minute of participation in the game and involve skating distances of 15–26 m. Such actions impose substantial demands on the locomotor and functional systems of ice hockey players which are accompanied by the accumulation of metabolites. Locomotion in ice hockey is largely based on gliding phases on the ice, resulting in a relatively large increase in the distance covered with relatively low mechanical work performed [5,6]. The greatest loads on the musculoskeletal and energy systems occur during acceleration and deceleration phases, which involve both concentric and eccentric high-force muscle contractions [7]. The ability to maintain rapid recovery between shifts, as well as within a single shift, reflects the effective interaction between aerobic and anaerobic energy systems.

Muscle oxygen uptake begins within seconds of intense exercise, reaching maximal values after approximately 45 s [8]. This duration is nearly twice as long as the typical shift in modern ice hockey. Consequently, achieving peak oxygen uptake results from the cumulative effect of repeated high-intensity efforts [5]. Studies [9,10] demonstrate that phosphocreatine (PCr) resynthesis constitutes the primary source of ATP during short-duration efforts and is not directly dependent on oxidative metabolism. It is supported by mitochondrial ATP production which becomes increasingly intensified during the sprinting exercise. Studies show [11,12,13,14] that oxidative phosphorylation is progressively activated during repeated efforts. In a single 6 s sprint, the contribution of oxidative phosphorylation does not exceed 10% of total energy provision. When sprinting exercises are repeated many times, the share of oxidative phosphorylation increases to 40–50%, while glycolytic metabolism is concurrently reduced [11]. The study demonstrated substantial PCr depletion following ice hockey players match play [15]. The ability of ice hockey players to repeatedly perform high-intensity, COD (change of direction) efforts is related with the kinetics of PCr resynthesis. After high-intensity efforts, a rapid PCr recovery is observed during the initial phase (30–60 s) followed by a slower stabilization phase (4–5 min) [9,10]. This mechanism allows for rapid recovery during rest intervals between game accesses [16,17,18].

Studies examining the influence of aerobic capacity level on the rate of PCr resynthesis do not provide conclusive findings to this matter. Bogdanis et al. [13] observed a relationship between the percentage of VO_2max_ at the lactate threshold and the rate of PCr resynthesis following sprint exercise. In contrast, McCully et al. [19] observed an acceleration of PCr recovery kinetics after a 14-day endurance training program. Similar findings have also been found in studies by McCully et al., Takahashi et al. and Laurent et al. [19,20,21]. In contrast, Cooke et al. [22] concluded that VO_2max_ is not a strong predictor of PCr recovery. The lack of a relationship between VO_2max_ and the rate of PCr resynthesis may be attributable to the limited variability of VO_2max_ values within this group of athletes, who obtained high aerobic capacity (55–60 mL·kg^−1^·min^−1^) [3,23,24].

Ice hockey players are not the only group in which variability has been observed in the strength of the relationship between VO_2max_ and the rate of PCr resynthesis. Athletes from disciplines such as female netball, basketball, field hockey [25], soccer [26] and rugby [27] demonstrate significant relationships between VO_2max_ and PCr recovery kinetics, whereas no such relationships have been confirmed in other sports, including Australian football [28], female field hockey [29], soccer [30] and basketball [31]. The inconsistency of findings linking VO_2max_ with performance during repeated short-duration, high-power efforts highlights the complex energetic nature of RHIE. Research [32,33] have shown that the ability to repeatedly perform high-speed efforts significantly differentiates elite ice hockey players. However, these studies do not fully explain the underlying mechanisms responsible for this differentiation. One potential factor may be the rate of PCr resynthesis during recovery periods between repeated sprint efforts, which is largely dependent on the capacity of aerobic metabolism [20,34]. Evidence from research conducted in other sports further indicates that substantial differences in repeated sprint performance may occur despite comparable VO_2max_ values [35,36]. Notably, the existing literature lacks studies examining the relationship between aerobic capacity expressed by VO_2max_ and oxygen uptake at metabolic thresholds in relation to the ability to repeatedly perform high-speed efforts characteristic of ice hockey.

The primary aim of this study was to determine the energetic structure of repeated high-intensity on-ice sprint exercise in ice hockey players by quantifying the relative contributions of the oxidative, glycolytic and ATP–PCr energy systems.

A secondary aim was to investigate the relationships between these energetic parameters and indicators of aerobic capacity, including directly measured VO_2max_, as well as oxygen uptake expressed at the aerobic and anaerobic thresholds.

Additionally, the study sought to evaluate the consistency between directly measured and estimated VO_2max_ values obtained using the 30–15 Intermittent Ice Test (30–15_IIT_) in a homogeneous group of trained ice hockey players.

It was hypothesized that repeated high-intensity on-ice sprint exercise in ice hockey players would be characterized by a substantial contribution of the ATP–PCr and oxidative energy systems, and that parameters describing the oxidative contribution and ATP–PCr resynthesis would be positively associated with aerobic fitness indicators, including VO_2max_ and oxygen uptake at the aerobic and anaerobic thresholds.

## 2. Materials and Methods

### 2.1. Study Design

The study was designed as an observational, exploratory, correlational investigation conducted in a semi-professional, homogeneous cohort of ice hockey players.

The study was conducted in a group of advanced 18-year-old ice hockey players who were members of the national team. All participants followed the same training and competition program under the supervision of a consistent coaching staff throughout a 10-month training period. Measurements were performed during a one-week break between the end of the domestic league season and the beginning of the preparatory phase for the World Championships.

All players consumed standardized meals with a balanced macronutrient composition, including carbohydrates, fats, and proteins. No nutritional supplementation was permitted during the 72 h preceding the study and throughout the completion of the second day of testing.

Participants were fully informed about the purpose of the study and the procedures involved. Prior to testing, they attended a practical on-ice demonstration of all test protocols.

### 2.2. Participants

Fourteen male semi-professional ice hockey players (body mass: 76.14 ± 7.13 kg; height: 181.5 ± 6.7 cm; BMI: 23.1 ± 1.6; experience in ice hockey training: 11 ± 1 years) from the Sports School of the National Ice Hockey Federation participated in this study. All participants provided written informed consent prior to participation in the study and were at least 18 years of age on the day of testing. They were in good health and reported no injuries and infections at the time of the study. The study was conducted in accordance with the Declaration of Helsinki [37] and approved by the ethical committee of Jan Dlugosz University in Czestochowa (code: KE-U/87/2025). Given the exploratory nature of the study and the elite, homogeneous sample, a sample size of fourteen players was deemed acceptable. A post hoc power analysis (α = 0.05, two-tailed) indicated that this sample provided approximately 80% statistical power to detect large correlations (|r| ≥ 0.69).

### 2.3. Procedure

The study followed a structured four-day on-ice protocol, with the daily testing and training procedures described in detail below.

Day 1. A low-intensity, on-ice tactical training session was conducted.

Day 2. The 30–15_IIT_ was performed. The test was conducted in pairs matched according to the distance covered during a 30–15_IIT_ completed prior to the competitive season. This pairing strategy was used to maximize the likelihood of maintaining consistent pairings throughout the test. Skating in pairs was intended to enhance motivation and promote maximal effort under progressively increasing fatigue.

Day 3. An active recovery session was implemented, consisting of aerobic-based functional training.

Day 4. RHIE on-ice was conducted. The test was performed using the same pairings as those applied during the 30–15_IIT_.

All exercise testing was conducted at an indoor, full-size ice rink compliant with International Ice Hockey Federation (IIHF) regulations. The tests were conducted under specific environmental conditions. The ice surface temperature was −4.7 ± 0.5 °C, while the air temperature above the ice field was 8.6 ± 0.4 °C. Additionally, the air temperature measured at the players’ bench, located 1.5 m from the ice surface, was 12.5 ± 1.5 °C. The ice surface was resurfaced after every three pairs of participants during both the 30–15_IIT_ and the RSE to ensure consistent testing conditions.

### 2.4. Physiological Measurements

Physiological parameters were recorded for 3 min prior to exercise in a standing position, throughout the entire exercise and for 15 min during post-exercise recovery in a seated position. Respiratory gas exchange was measured breath-by-breath using portable K5 metabolic analyzers (Cosmed, Rome, Italy). Capillary blood samples were obtained before and immediately after exercise to determine blood lactate concentration. Heart rate was continuously monitored throughout all tests and rest periods using the Polar Team Pro system (Polar, Kempele, Finland). The energetic contributions of the specific ice hockey test were determined from breath-by-breath oxygen uptake and blood lactate data, analyzed with OriginPro 2025 software (OriginLab Corporation, Northampton, MA, USA). Based on the physiological responses obtained during the 30–15_IIT_, indicators of aerobic fitness were calculated, including maximal values and values corresponding to the aerobic (AeT) and anaerobic (AnT) thresholds. Based on the dynamics of physiological indicator values recorded during the 30–15_IIT_ test, the following were calculated for each subject: VO_2max_ value, and VO_2_ values at the AeT and AnT metabolic thresholds. The V-slope method was used to determine VO_2_ at the metabolic thresholds [38]. The AeT threshold (VT_1_) corresponds to the work intensity at which VE begins to increase disproportionately to oxygen uptake (VO_2_). The AnT threshold (VT_2_) corresponds to the work intensity at which VCO_2_ values begin to increase at a faster rate than VO_2_.

### 2.5. The 30–15 Intermittent Ice Test (30–15_IIT_)

The 30–15_IIT_ was used to determine VO_2max_ as well as the percentages of VO_2max_ at the aerobic (AeT) and anaerobic (AnT) thresholds, due to its movement structure exercise conditions. Since the energy structure of an ice hockey player’s specific effort was determined during an on-ice exercise, it was methodologically appropriate to determine the aerobic capacity using an on-ice testing protocol. One of the main goals of the study was to examine the relationship between the level of aerobic capacity and the energy structure of effort reflecting the specific load of a hockey player during match play.

**Figure 1:**
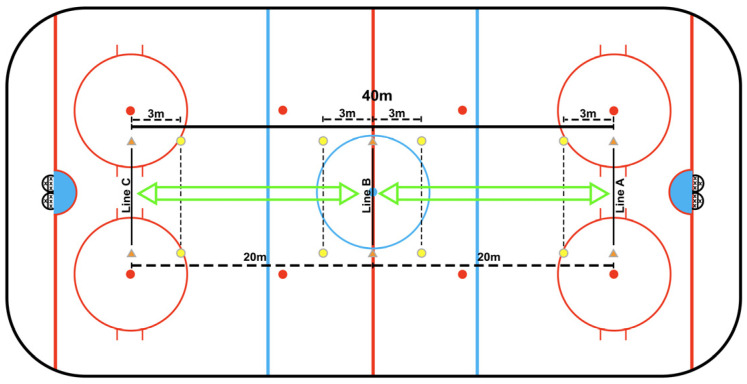
On-ice protocol of the experiment 30–15 Intermittent Ice Test (30–15_IIT_).

The protocol and zones configuration of the 30–15_IIT_ were identical to those of the original 30–15 Intermittent Fitness Test (30–15_IFT_), with the exception that skating velocity increased by 0.63 km/h at each 30 s stage, beginning at an initial velocity of 10.8 km/h. Each effort was separated by a 15 s passive recovery period. At the end of each shuttle, players were required to come to a complete full stop within the designated line before changing direction, ensuring consistent movement patterns throughout the protocol (Figure 1). The skating pace was determined by a pre-recorded beep signal. The same criteria as those in 30–15_IFT_ were used to determine the end of the test. The velocity at the last fully completed stage was recorded as V_max_ (maximal skating velocity) [39].

### 2.6. Estimation of Maximal Oxygen Uptake from Velocity of the 30–15_IIT_

The purpose of estimating VO_2max_ using the 30–15_IIT_ procedure was to determine the consistency between measured and estimated values.

Because the testing procedure was validated on a group of hockey players with varying levels of aerobic capacity and the study group was homogeneous in terms of training preparation, an additional analysis was attempted to determine whether the exercise test conditions yielded comparable individual VO_2max_ values when determined using both direct measurement and estimation procedures for each player.

Estimated VO_2max_ was calculated from the maximal skating velocity reached during the 30–15_IIT_ according to the following equation [40]:

(1) VO_2max_ = 28.3 − (2.15 × G) − (0.741 × A) − (0.0357 × BM) + (0.0586 × A × V_max_) + (1.03 × V_max_)

where VO_2max_ is maximal oxygen uptake [mL/kg/min]; G is gender (female = 2, male = 1); A is age [years]; BM is body mass [kg]; V_max_ is the maximal skating velocity of the 30–15_IIT_ [km/h]; 28.3, 2.15, 0.741, 0.0357, 0.0586, and 1.03 are constant coefficients.

### 2.7. The Repeated High-Intensity Effort (RHIE) On-Ice

The literature has focused on the need to adapt the structure of exercise test protocols in ice hockey to the physiological and motor requirements of the game [41]. A work-to-rest interval of 15 s of effort followed by 15 s of recovery represents an organizational structure commonly applied in exercise speed training in ice hockey [42].

The specific effort protocol derived from the on-ice Repeated Ability Test (RAT) was used to reproduce Repeated High-Intensity Effort (RHIE) assessment of ice hockey performance, incorporating both offensive (forward skating and changes of direction) and defensive (backward skating) movement patterns. This test has been shown to replicate the multidirectional and intermittent demands characteristic of competitive ice hockey [43].

**Figure 2:**
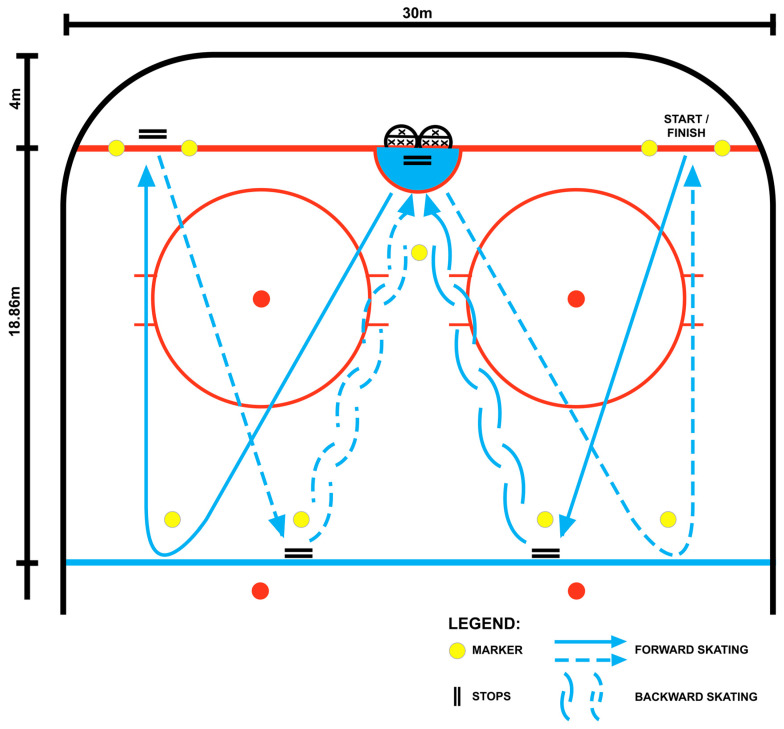
On-ice protocol for the experiment Repeated High-Intensity Effort (RHIE) based on Repeated Ability Test (RAT).

The RAT protocol consisted of an 18 m forward sprint to a full stop, followed by 22 m of backward skating to a full stop at the goal line. Participants then performed 22 m of forward skating ending with a sharp turn, and completed the sequence with an additional 18 m forward sprint to the finish line (Figure 2). Each work interval lasted approximately 15 s, followed by 15 s of passive recovery. The sequence was performed 3 times per set, across 3 sets in total, with a 90 s rest period between sets.

### 2.8. Calculations of Energy System Contribution—PCr-La-O_2_

The contributions of the oxidative, glycolytic and ATP-PCr systems were determined by measuring oxygen consumption, blood lactate accumulation and the fast component of excess post-exercise oxygen consumption (EPOC_FAST_) [44].

Breath-by-breath metabolic gas analysis was used to measure oxygen uptake during resting period, throughout the exercise protocol and the 15 min post-exercise recovery phase.

Baseline oxygen consumption (VO_2 BASELINE_) was calculated by multiplying the equivalent of VO_2_ in a standing position 4.5 [mL·kg^−1^·min^−1^] [45,46] and the total duration of the effort.

(2) VO_2 BASELINE_ = 4.5 mL·kg^−1^·min^−1^ × Total effort time

Exercise oxygen consumption (VO_2 EXERCISE_) was quantified as the area under the VO_2_ curve using the trapezoidal method. The oxidative (aerobic) contribution to energy expenditure (W_AER_) was then calculated by subtracting baseline oxygen consumption from exercise oxygen uptake [47,48]:

(3) W_AER_ = VO_2 EXERCISE_ − VO_2 BASELINE_

The glycolytic contribution (W_BLC_) was estimated based on the assumption that an increase of 1 [mmol·L^−1^] in blood lactate above baseline corresponds to an oxygen equivalent of 3 [mL·kg^−1^·mmol^−1^·L].

The difference between peak and baseline lactate concentrations (ΔLa) was multiplied by this oxygen equivalent and by the athlete’s body mass. The resulting oxygen equivalent [mL·kg^−1^] was then converted to liters and subsequently to energy (kJ), assuming a caloric equivalent of 20.92 kJ per liter of O_2_:

(4) W_BLC_ = (La _MAX_ − La _BASELINE_) × 3 × 20.92 × BM × 0.001

where W_BLC_ is glycolytic energy system contributions [kJ], La _MAX_ is peak blood lactate concentration [mmol·L^−1^], La _BASELINE_ is pre-exercise blood lactate concentration [mmol·L^−1^], 3 [mL·kg^−1^·mmol^−1^·L] is the lactate oxygen equivalent, 20.92 is the caloric equivalent of oxygen, [J·mL^−1^], BM is body mass [kg], 0.001 is the joule-to-kilojoule conversion factor.

The anaerobic alactic contribution (W_PCr_), representing the ATP-PCr metabolism pathway, was considered as the fast component of excess post-exercise oxygen consumption (EPOC_FAST_). Post-exercise VO_2_ kinetics were modeled using a mono- or bi-exponential function fitted in OriginPro 2025 (OriginLab Corporation, Northampton, MA, USA).

(5) VO_2(t)_ = VO_2 BASELINE_ + A_1_[e^−(t−δ)/τ^_1_] + A_2_[e^−(t−δ)/τ^_2_]

where VO_2(t)_ is oxygen uptake at time t, VO_2 BASELINE_ is baseline oxygen uptake, A_1_ and A_2_ are the amplitudes of the fast and slow components, δ is the time delay, and τ_1_ and τ_2_ are the corresponding time constants [49].

The fast component of EPOC was calculated as:

(6) EPOC_FAST_ = A_1_ × τ_1_

Total energy expenditure was obtained as the sum of oxidative, glycolytic, and ATP–PCr system contributions. Each energy system’s contribution was subsequently expressed as a percentage of total energy expenditure [45].

### 2.9. Statistical Methods

Mean, standard deviation (SD) and 95% confidence interval (CI) were used to represent the average and the typical spread of values of all the measured variables. The normality of the data distribution was verified using the Shapiro–Wilk test. Pearson’s correlation coefficient (r) determined the relationships between associated variables. Correlation coefficients were visualized using correlograms (heatmaps). In these graphical representations, the direction of the relationships was indicated by color: red represented positive correlations (r > 0) and blue represented negative correlations (r < 0). The strength of the correlation was expressed by color intensity, with darker shades indicating stronger relationships. Thresholds for statistical significance (*p*-values) were provided below each correlogram. Calculated correlation coefficients were not adjusted for multiple comparisons. Statistical significance was set at α ≤ 0.05. All the statistical analyses were performed with OriginPro 2025 (OriginLab Corporation, Northampton, MA, USA).

## 3. Results

### 3.1. Results of the 30–15 Intermittent Ice Test (30–15_IIT_)

Descriptive statistics for all variables recorded during the 30–15_IIT_, characterizing the aerobic capacity profile of the studied ice hockey players, are presented in Table 1.

**Figure 3:**
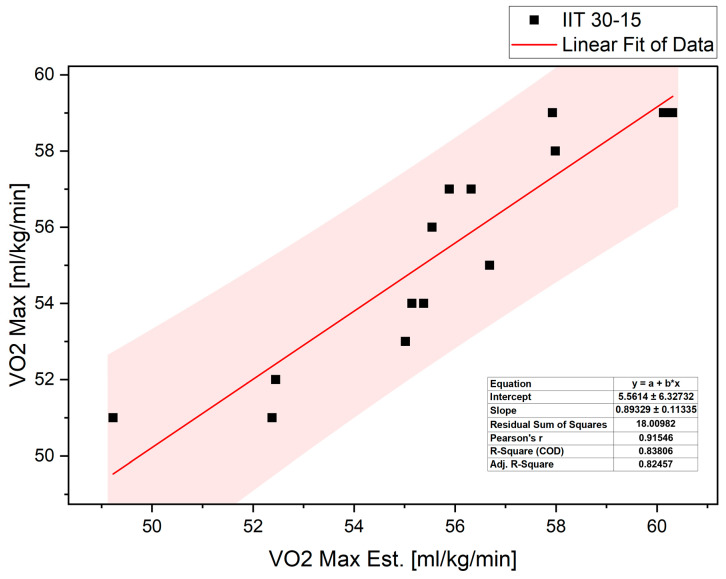
Relationship between directly measured VO_2max_ and VO_2max_ estimated from the 30–15 Intermittent Ice Test (30–15_IIT_) in ice hockey players.

The regression coefficients between VO_2max_ estimated using the 30–15 test formula and VO_2max_ measured directly are presented in Figure 3.

Black squares represent individual data points, while the solid red line indicates the linear regression fit, with the shaded area showing the 95% confidence interval. A strong relationship was observed between estimated and measured VO_2max_ (Pearson’s r = 0.915), with the regression model explaining a large proportion of variance (R^2^ = 0.838; adjusted R^2^ = 0.825). The regression equation parameters and goodness-of-fit statistics are presented within the figure. The coefficient of determination (R^2^ = 0.838) indicates that approximately 83% of the variance in VO_2max_ values estimated using the 30–15_IFT_ algorithm was explained by the VO_2max_ values measured directly during exercise.

### 3.2. Results of the Repeated High-Intensity Effort (RHIE) On-Ice

Descriptive statistics for work time variables and blood lactate concentration characterizing performance in the RHIE on-ice protocol performed by the studied group of ice hockey players are presented in Table 2.

The analysis of performance times across the 3 RHIE series demonstrated a stable level of performance in the studied ice hockey players, as reflected by comparable mean completion times. The absence of a significant increase in time between consecutive series indicates a high capacity to maintain power output during repeated, high-intensity on-ice exercise. The applied recovery periods of 90 s between series and 15 s between repetitions were sufficient to restore energy reserves in the majority of participants, allowing subsequent efforts to be performed at an intensity comparable to the preceding one. At the same time, the high increase in blood lactate concentration (∆La: 8.68 ± 1.59 mmol·L^−1^) indicates a substantial contribution of anaerobic glycolysis as a supplementary energy source.

Descriptive statistics of energy expenditure variables recorded during RHIE on-ice in the studied group of ice hockey players are presented in Table 3.

The total energy expenditure during RHIE in the studied group of ice hockey players averaged 562.0 ± 52.0 kJ, corresponding to 7.40 ± 0.53 kJ·kg^−1^ of body mass. The distribution of energy sources was characterized by a dominant contribution of aerobic metabolism, which accounted for 63.1 ± 2.6% of the total work and corresponded to an expenditure of 352.8 ± 33.8 kJ (4.64 ± 0.31 kJ·kg^−1^). The contribution of anaerobic glycolysis (lactic pathway) was substantially lower, amounting to 7.4 ± 1.5%, which corresponded to 41.2 ± 8.4 kJ (0.54 ± 0.10 kJ·kg^−1^). Phosphagen metabolism (anaerobic alactic pathway) accounted for 29.8 ± 2.9% of the generated energy, translating into an expenditure of 167.9 ± 24.9 kJ (2.22 ± 0.34 kJ·kg^−1^).

### 3.3. Correlation of Physiological Parameters

**Figure 4:**
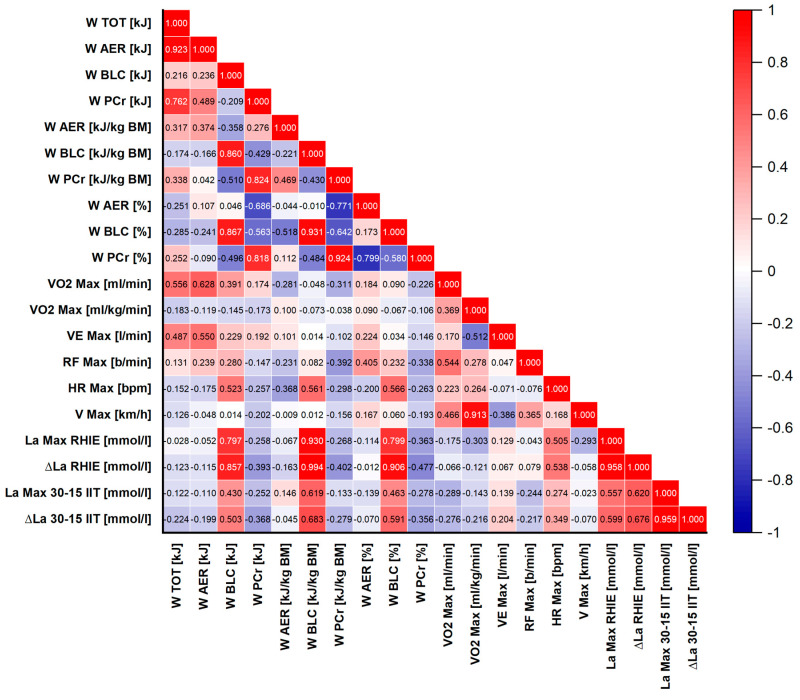
Heatmap of Pearson’s correlations between performance indices from the Repeated High-Intensity Effort (RHIE) on-ice and maximal aerobic capacity parameters from the 30–15 Intermittent Ice Test (30–15_IIT_) (*p* ≤ 0.05 at r ≥ 0.532; *p* ≤ 0.001 at r ≥ 0.790).

To assess the relationships between ice hockey players’ performance during high-intensity intermittent on-ice exercise and the metabolic profile of this effort, as well as maximal aerobic capacity parameters, Pearson’s correlation analysis was performed between key variables obtained from the RHIE and the 30–15_IIT_. The results are visualized using a heatmap (Figure 4).

The correlation analysis revealed no statistically significant relationships between maximal oxygen uptake (VO_2max_), expressed in absolute [mL·min^−1^] and relative terms [mL·kg^−1^·min^−1^], and energy expenditure indices (W_AER_, W_BLC_, W_PCr_, W_TOT_), work time variables (T_S1_, T_S2_, T_S3_, T_TOT_), or markers of anaerobic lactic metabolism (La_Max_ RHIE, ∆La RHIE) obtained during RHIE. The only exception was a moderate correlation between absolute VO_2max_ and absolute aerobic work performed during RHIE (W_AER_ [kJ]; r = 0.63, *p* = 0.016).

Similarly, estimated VO_2max_ values (VO_2max_ Est.) and velocity at VO_2max_ (V_max_) showed no significant relationships with RHIE parameters. Total time (T_TOT_) was, however, very strongly correlated with the times recorded in each of the RHIE on-ice series (T_S1_, T_S2_, T_S3_; r > 0.991, *p* < 0.001 in all cases), confirming high internal consistency and reliability of the test.

These relationships are expected, as the W_BLC_ component is derived from changes in blood lactate concentration. Thus, athletes exhibiting a greater relative contribution of anaerobic glycolysis were characterized by higher lactate accumulation.

Work times in individual RHIE on-ice series were not significantly correlated with any lactate-related indices, confirming the minor role of this metabolic component in determining performance time during high-intensity intermittent skating. A significant correlation was found between peak blood lactate concentration after RHIE on-ice (La_Max_ RAT) and peak lactate concentration following the 30–15_IIT_ (La_Max_ 30–15_IIT_; r = 0.56, *p* = 0.038). Likewise, strong correlations were observed between lactate increments in both tests (∆La RAT and ∆La 30–15_IIT_; r = 0.68, *p* = 0.008).

Importantly, lactate increment during the 30–15_IIT_ (∆La 30–15_IIT_) was also moderately correlated with the percentage contribution of lactic work in the RHIE on-ice (W_BLC_ [%]; r = 0.59, *p* = 0.026). Internal relationships within the energy structure of the RHIE on-ice were further confirmed: the percentage contribution of aerobic work (W_AER_ [%]) was strongly correlated with the percentage contribution of phosphagen work (W_PCr_ [%]; r = −0.80, *p* = 0.0006), emphasizing the compensatory nature of these energy pathways.

As expected, total energy expenditure during the RHIE on-ice (W_TOT_ [kJ]) was very strongly correlated with absolute aerobic work (W_AER_ [kJ]; r = 0.92, *p* < 0.0001). In addition, the durations of all RHIE on-ice series were strongly interrelated (r > 0.97, *p* < 0.0001), further confirming the high reliability of the test. No significant correlations were observed between maximal heart rate (HR_Max_) or maximal ventilation (VE_Max_) obtained during the 30–15_IIT_ and the RHIE on-ice energy expenditure variables.

Overall, the results indicate that within this relatively homogeneous group of players, maximal aerobic capacity (VO_2max_) did not show statistically significant relationships with the ability to perform repeated high-intensity on-ice work, as assessed by the RHIE on-ice as well as its metabolic cost. The key factor differentiating physiological responses during the RHIE on-ice appears to be the propensity to use anaerobic glycolysis, which strongly determines the degree of lactate accumulation. Moreover, individual tendencies toward high lactate production seem to be reproducible traits, as reflected by significant correlations between different exercise tests (the RHIE on-ice and 30–15_IIT_). The contribution of aerobic work was also moderately and significantly correlated with both absolute (W_PCr_ [kJ]; r = −0.686, *p* = 0.007) and relative (W_PCr_ [kJ·kg^−1^]; r = −0.771, *p* = 0.001) phosphagen work.

**Figure 5:**
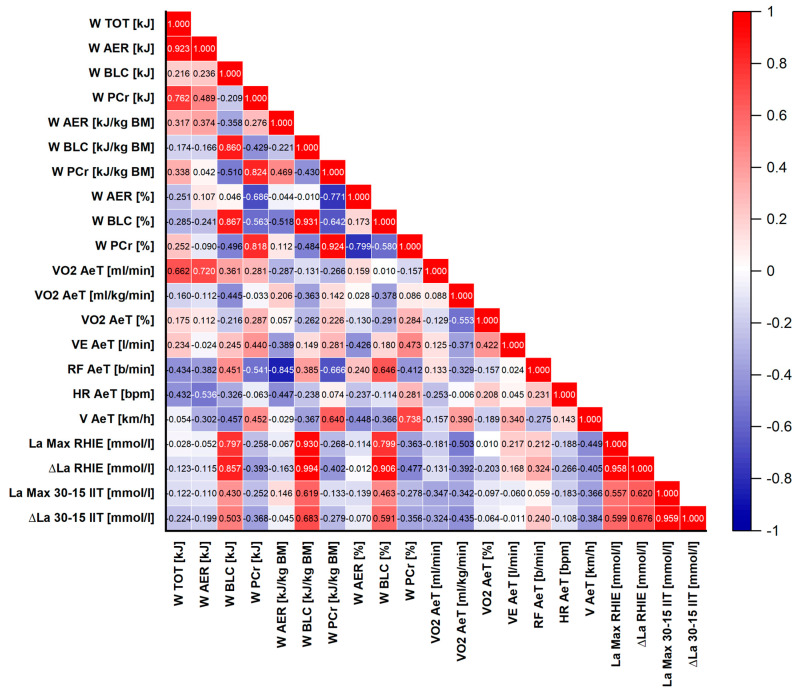
Heatmap illustrating Pearson’s correlations between performance parameters of the Repeated High-Intensity Effort (RHIE) on-ice and aerobic capacity parameters recorded at the aerobic threshold (AeT) during the 30–15 Intermittent Ice Test (30–15_IIT_). (*p* ≤ 0.05 at r ≥ 0.532; *p* ≤ 0.001 at r ≥ 0.790).

**Figure 6:**
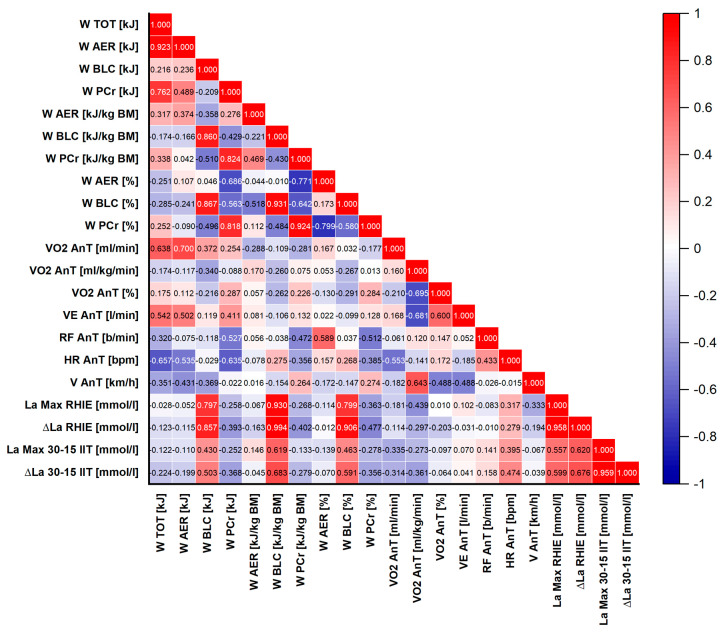
Heatmap illustrating Pearson’s correlations between performance parameters of the Repeated High-Intensity Effort (RHIE) on-ice and aerobic capacity parameters recorded at the anaerobic threshold (AnT) during the 30–15 Intermittent Ice Test (30–15_IIT_). (*p* ≤ 0.05 at r ≥ 0.532; *p* ≤ 0.001 at r ≥ 0.790).

To provide a comprehensive assessment of the relationships between ice hockey players’ performance during high-intensity intermittent on-ice exercise, the metabolic profile of this effort, and aerobic capacity, Pearson’s correlation analysis was extended to include relationships between key parameters of RHIE on-ice and threshold—the aerobic (AeT) and anaerobic (AnT) thresholds—obtained from the 30–15_IIT_. The results are visualized using heatmaps (Figure 5 and Figure 6).

Parameters measured at the first ventilatory threshold (AeT) showed weak and predominantly non-significant correlations with performance indices obtained in the RHIE on-ice as with the energetic structure of this exercise. The only exception was a statistically significant correlation between absolute oxygen uptake at the aerobic threshold (VO_2_ AeT [mL·min^−1^]) and absolute aerobic work during the RHIE on-ice (W_AER_ [kJ]; r = 0.720, *p* = 0.004). Most AeT-derived variables—including oxygen uptake, minute ventilation, heart rate and breathing frequency—formed a distinct cluster exhibiting minimal and non-significant relationships with parameters of anaerobic performance and metabolic power.

Performance indices determined at the anaerobic threshold (AnT) demonstrated more heterogeneous and more frequently significant relationship compared with those observed at the AeT. A significant correlation was found between absolute oxygen uptake at AnT (VO_2_ AnT [mL·min^−1^]) and absolute aerobic work (W_AER_ [kJ]; r = 0.700, *p* = 0.005), as well as total work (W_TOT_ [kJ]; r = 0.638, *p* = 0.014). Relative oxygen uptake at AnT (VO_2_ AnT [mL·kg^−1^·min^−1^]) was significantly correlated with its percentage value (VO_2_ AnT [%]; r = −0.695, *p* = 0.006) and minute ventilation (VE AnT [L·min^−1^]; r = −0.681, *p* = 0.007), while showing a relationship with movement velocity at this threshold (V AnT [km·h^−1^]; r = 0.643, *p* = 0.013). Heart rate at AnT (HR AnT [bpm]) exhibited significant correlations with both absolute oxygen uptake (VO_2_ AnT [mL·min^−1^]; r = −0.553, *p* = 0.040) and total metabolic work (W_TOT_ [kJ]; r = −0.657, *p* = 0.011). In contrast to AeT-derived variables, AnT parameters showed moderate, statistically significant relationships with absolute indices of metabolic work (W_AER_ [kJ], W_TOT_ [kJ]) and formed a distinct cluster including VO_2_ AnT [mL·kg^−1^·min^−1^], VE AnT [L·min^−1^], and V AnT [km·h^−1^]. Notably, breathing frequency at AnT (RF AnT [min^−1^]) demonstrated a unique and significant relationship with sprint times in the RHIE on-ice (r ≈ 0.66–0.70, *p* < 0.01), distinguishing it from other threshold-related variables.

## 4. Discussion

Both match play and specialized on-ice training in ice hockey constitute complex physical demands that engage all major energy systems [50]. Despite the long history of this sport and the growing body of laboratory and field-based research, several physiological determinants of ice hockey performance remain insufficiently elucidated. Among the most relevant issues are oxygen uptake kinetics during intermittent exercise and the role of maximal oxygen uptake (VO_2max_) in the resynthesis of PCr, the concentration of which is markedly reduced following each high-intensity involvement [23].

The primary aim of this study was to determine the energetic structure of repeated high-intensity on-ice sprint exercise in ice hockey players by quantifying the relative contributions of the oxidative, glycolytic and ATP–PCr energy systems.

A secondary aim was to investigate the relationships between these energetic parameters and indicators of aerobic capacity, including directly measured VO_2max_, as well as oxygen uptake expressed at the aerobic and anaerobic thresholds.

Additionally, the study sought to evaluate the consistency between directly measured and estimated VO_2max_ values obtained using the 30–15 Intermittent Ice Test (30–15_IIT_) in a homogeneous group of trained ice hockey players.

At high frequencies efforts, the duration of recovery periods appears to be insufficient for complete PCr resynthesis in a proportion of fast-twitch muscle fibers [15,51]. Consequently, rest [52,53,54,55,56] and energy restoration represent a specific challenge for the interaction of the involved energy systems. Previous studies [57,58] have demonstrated that the magnitude of fatigue differs substantially depending on the structure of the exercise. In ice hockey, skating requires frequent changes of direction performed at high intensity [2,59]. Prior studies [11,60,61,62] indicate that intermittent exercise or short-duration continuous exercise performed at supramaximal intensity results in a pronounced development of fatigue.

In the present study, during a hockey-specific exercise performed at maximal intensity, an attempt was made to quantify the contribution of the three main metabolic energy sources and to examine their relationships with indices of aerobic capacity. During an ice hockey match, aerobic metabolism is strongly activated, as evidenced by mean heart rate values reaching approximately 85% of HR_max_ [2,15,63]. A high aerobic power combined with rapid oxygen uptake kinetics may therefore reduce the contribution of anaerobic energy pathways to the overall energetic cost of hockey-specific exercise. This creates conditions that decrease disturbances in the intracellular environment and delay the development of fatigue [13,64,65]. The available literature [23,24] indicates that ice hockey players are characterized by a high level of aerobic capacity with VO_2max_ values typically ranging between 55 and 60 mL O_2_·kg^−1^·min^−1^. In the present study, the mean VO_2max_ reached 55 mL O_2_·kg^−1^·min^−1^, allowing this group to be considered representative of the aerobic capacity profile commonly observed in this sport.

However, there is still a lack of convincing evidence regarding the role of VO_2max_ magnitude in attenuating fatigue during repeated high-intensity exercise. Opinions regarding the role of aerobic capacity in sustaining the ability to perform repeated maximal-intensity efforts remain inconclusive [66,67,68]. In this investigation, no metabolic level disturbances were observed that would impair the athletes’ readiness to perform work at maximal intensity. The analysis of completion times across the three consecutive test series revealed a stable level of performance in the examined ice hockey players. The absence of a statistically significant increase in performance time between series indicates a high capacity to maintain power output during repeated, high-intensity on-ice efforts. The recovery intervals of 90 s between series and 15 s between repetitions were therefore sufficient to allow restoration of energy reserves. Aerobic metabolism reaches maximal intensity after approximately 45 s, which does not correspond to the typical characteristics of ice hockey match play. However, this response may be the result of the accumulation of repeated efforts separated by very short recovery periods [11,69].

In the studied group of ice hockey players, the distribution of energy sources throughout the entire effort performed according to the RHIE on-ice was characterized by a dominant contribution of aerobic metabolism. Aerobic processes accounted for 63.1 ± 2.6% of the total work performed, corresponding to an energy expenditure of 352.8 ± 33.8 kJ (4.64 ± 0.31 kJ·kg^−1^). The high contribution of aerobic metabolism to the overall energy balance during high-power exercise indicates that it is a primary factor determining PCr resynthesis between bouts of high-intensity effort [64,70]. Studies by Bangsbo [71] and Bangsbo et al. [69] indicate that muscle oxygen uptake begins within the first few seconds of intense exercise and accelerates during subsequent repetitions, a response characteristic of ice hockey activity. In the present study, no relationship was observed between skating speed during specific on-ice exercise and VO_2max_. This lack of relationship may be attributable to the complex nature of muscular oxidative capacity. Muscle oxidative capacity is determined by several factors, including mitochondrial content and function [35], maximal activity of oxidative enzymes [72] and capillary density [35,72,73]. In contrast, VO_2max_ is considered primarily a marker of the body’s capacity for oxygen transport [74]. Numerous studies—such as Rampinini et al. [26,75], Christensen et al. [76], Dupont et al. [77,78]—have demonstrated a close relationship between oxygen uptake kinetics and performance during exercise tests involving both high-intensity intermittent sprints and repeated sprint efforts. The delay in achieving maximal muscle VO_2_ activation results in a greater contribution of anaerobic ATP supply at the onset of each shift and during sudden fluctuations in exercise intensity [8,50]. These conditions were met by the specific exercise protocol applied in the present study. The findings of this investigation are consistent with those observed by Love [79] who found no significant relationship between maximal aerobic power and the effectiveness of hockey-specific work. Similarly, Bishop et al. [29] found no significant relationship between maximal aerobic power and repeated sprint performance in elite female ice hockey players. In contrast, studies by Gaitanos et al. [11], Parolin et al. [12], Bogdanis et al. [13], and Serresse [14] have emphasized the connection of a relationship between the progressive activation of aerobic metabolism and oxidative phosphorylation. During 6 s sprint effort, the contribution of oxidative phosphorylation is limited (up to ~10%); however, during a series of repeated efforts, this contribution increases reaching approximately 40–50% of the total energy supply.

In this research, the contribution of energy derived from anaerobic alactic metabolism amounted to 29.8 ± 2.9%, corresponding to an energy expenditure of 167.9 ± 24.9 kJ (2.22 ± 0.34 kJ·kg^−1^). Depletion and prolonged recovery of PCr and ATP concentrations in fast-twitch muscle fibers may represent a limiting factor for the ability to maintain performance during repeated high-intensity actions [18,80,81,82]. Previous studies [11,12,13,16,60,83] indicate that PCr stores may be substantially reduced (by approximately 50–75%) after 6–10 s of maximal work. Therefore, both during match play and training, ice hockey players require continuous restoration of PCr stores during short periods of complete or relative recovery [3]. In a study conducted by Casey et al. [80], performance during successive bouts of high-intensity exercise was shown to be associated with the rate of PCr resynthesis. Similarly, Bogdanis et al. [13] observed strong correlations between the proportion of resynthesized PCr and the degree of restoration of peak power output after 90 and 180 s of recovery. During a 6 s maximal effort, ATP is supplied in nearly equal proportions from PCr and glycolysis. Both processes are activated immediately after starting the exercise, with glycolysis reaching near-maximal rates within the first 5–6 s [11,12,14,83]. However, the continuation of very high-intensity work results in a reduction in glycolytic rate and an increased contribution of oxidative metabolism [11,12,83].

Balsom et al. [84] demonstrated that increase in VO_2max_ is associated with reduced blood lactate accumulation during high-intensity exercise. Reduced oxygen availability impairs exercise performance and increases blood lactate concentration [85]. In the present study, mean blood lactate values measured after completion of the RHIE on-ice averaged 10.0 ± 1.62 mmol·L^−1^, with a mean lactate increment (∆La) of 8.68 ± 1.59 mmol·L^−1^. Blood lactate concentration reflects both the rate of lactate appearance and its removal. Consequently, blood lactate values do not always correspond to intramuscular lactate concentrations during intermittent exercise [86].

Decrease in intramuscular lactate concentration following high-intensity exercise is a slow process, lasting several minutes. In the present study, lactate measurements were conducted over a 15 min post-exercise period to determine the peak post-exercise lactate concentration. In the research the contribution of glycolytic metabolism to the total energy expenditure was the smallest among the three analyzed energy systems. It was 7.4 ± 1.5%, which corresponded to an energy expenditure of 41.2 ± 8.4 kJ (0.54 ± 0.10 kJ·kg^−1^). The post-exercise values clearly indicate a high level of glycolytic activity during the RHIE derived from the on-ice RAT. As the test protocol consisted of repeated bouts rather than a single exercise effort, it can be assumed that a partial restoration of glycolytic reserves occurred throughout the test.

In summary, based on the results of the present study and previous research, there appears to be no relationship between maximal aerobic power and ice hockey players’ performance during specific intermittent exercise within this relatively homogeneous group.

The high contribution of the PCr and O_2_ in the energy systems indicates that these two metabolic pathways are dominant in the execution of specific tasks during ice hockey play. Together, they account for more than 90% of the total energy generated during on-ice exercise. Keiner et al. [87] demonstrated that linear sprint performance explains approximately 15–59% of the variance in match performance. Thus, sprinting represents one of the fundamental performance actions determining game effectiveness in ice hockey. The results of the present study clearly indicate the need to develop a high level of aerobic metabolic capacity in athletes during the training process. The contribution of aerobic energy pathways during sprint efforts in ice hockey players is approximately 60% of the total energy supply.

Future research should examine the relationship between maximal aerobic power and recovery process following multiple simulated exercise bouts, as well as the stability of the energetic structure of specific on-ice exercise in ice hockey players across the competitive season.

## 5. Conclusions

The present study provides an image of the relationships between maximal aerobic capacity, the ability to perform repeated high-intensity work and the body’s metabolic response in ice hockey players.

The primary finding of this study is the absence of significant correlations between maximal oxygen uptake (VO_2max_) and repetitive exercise parameters, including total work time, the input of energy system contributions and lactate accumulation. This suggests that in the context of short-duration, repeated on-ice sprints, traditionally understood maximal aerobic capacity may not be the primary limiting factor in the ability to sustain power output.

The results clearly indicate that individual responses in the RHIE on-ice are strongly determined by the propensity to utilize anaerobic glycolysis and individual tolerance to metabolic acidosis. Correlations observed between lactate indices from two different tests (RHIE on-ice and 30–15_IIT_) suggest that a propensity for high lactate accumulation may be a relatively stable, individual metabolic characteristic expressed across different exercise protocols.

Differential role of metabolic thresholds. Correlation analysis revealed a clear difference in the importance of the first (AeT) and second (AnT) ventilatory thresholds for assessing exercise capacity. Parameters at the aerobic threshold (AeT) showed minimal relationship with indicators of anaerobic performance, remaining in an isolated cluster. However, indicators at the anaerobic threshold (AnT), particularly absolute oxygen uptake (VO_2_ AnT [mL/min]) and velocity (V AnT), showed significant correlations with absolute aerobic and total workload. This suggests that performance at the AnT, which is an indicator of high-intensity work capacity with a dominant aerobic metabolism, may be more predictive of the potential for intensive metabolic work than VO_2max_ alone.

In summary, the obtained data indicate that in a population of 18-year-old ice hockey players, the ability to repeatedly perform specific high-intensity work may be largely independent of VO_2max_. The resulting image of the relationship between VO_2max_ and the energy structure of repeated sprinting efforts on-ice may be a result of limiting this indicator’s information to oxygen transport capacity. Oxygen uptake kinetics, mitochondrial density, and threshold performance may be more functionally relevant to the performance of maximal intensity efforts. The key factors differentiating young ice hockey players in terms of their ability to perform repeated sprinting efforts include: individual energy pathway utilization profile (particularly glycolytic propensity), the ability to work at the second ventilatory threshold (AnT), and individual tolerance to the accumulation of anaerobic glycolytic metabolites.

## 6. Limitations

Although the present study provides valuable insights into the energetic structure of specific on-ice exercise in ice hockey players and the relationships between energy profile variables and performance during intermittent sprint efforts performed on-ice, limitations should be acknowledged.

A primary limitation relates to the method used to determine the energetic structure of specific ice hockey efforts. A comparative analysis of methods conducted by Ambaum and Hoppe [88] identified the maximal accumulated oxygen deficit (MAOD) method as the most accurate approach for estimating the energetic cost of effort. However, the PCr–La–O_2_ method remains the only approach that takes into account the two components of anaerobic work that are critical in ice hockey performance. Kaufmann et al. [45] and Luches-Pereira et al. [89] found moderate measurement validity of this method for determining energetic structure. In contrast, Hatauta et al. [90], Valenzuela et al. [91], and Hill et al. [92] demonstrated high measurement validity using the same approach. It should also be noted that the excess post-exercise oxygen consumption (EPOC) method does not constitute as a direct measurement method. During intermittent exercise, the slow component phase cannot be identified between bouts. Within short recovery intervals, in the fast component phase, oxygen uptake increases as a consequence of the beginning of the subsequent effort. Therefore, results obtained using this method should be interpreted with caution, particularly with respect to the conclusiveness of the derived inferences. The estimation of the energy contribution derived from anaerobic glycolysis is also subject to limitations. High lactate clearance may lead to an underestimation of the contribution of anaerobic glycolysis to exercise energetics in this applied method.

Due to the exploratory nature of the correlation analysis and the small sample size, adjustments for multiple comparisons were not applied. Therefore, the absence of statistically significant correlations should be interpreted cautiously, as the study may be underpowered to detect moderate relationships. We acknowledge that the sample size was limited, as only 14 ice hockey players aged 18 years participated in the present study. However, this was a deliberate methodological approach aimed at ensuring sample homogeneity. All participants followed the same training program, which minimized the influence of potential confounding factors such as environmental conditions, dietary habits, the volume and structure of training loads and coach-related individual differences. The measurements were conducted during a period of high competitive readiness of the participants. Consequently, the results do not allow for generalization of the determined energetic structure to other phases of the annual training cycle. The relationship between the recorded indicators of specific exercise and maximal aerobic power (VO_2max_) therefore remains an open question. It can be hypothesized that during the preparatory period or phases characterized by a lower volume of specialized on-ice training, such relationships might become more apparent.

The transferability of the study findings and the resulting conclusions to other training groups is limited. This limitation may be particularly pronounced in younger athletes, whose anaerobic capacity systems are still subject to developmental constraints, as well as in female players, who differ from males in hormonal status. Caution should also be considered when extrapolating the study findings to athletes with large training experience. In such individuals, both the energetic structure of exercise and VO_2max_ values may be influenced by the level of movement technique during specific on-ice drills as well as during the 30–15_IIT_ test.

In summary, the scope of limitations resulting from participant selection and the method used to determine exercise energetic structure necessitates a cautious interpretation of the results.

## Figures and Tables

**Table 1:** Values of selected physiological variables obtained at maximal intensity and at the aerobic (AeT) and anaerobic (AnT) metabolic thresholds in ice hockey players during the 30–15 Intermittent Ice Test (30–15_IIT_).

Parameter	Intensity	M ± SD	±95% CI of M	CV
VO_2_ [mL/min]	Max	4211.8 ± 424.08	(3966.9; 4456.6)	10.07
	AnT	3237.2 ± 301.63	(3063.1; 3411.4)	9.32
	AeT	2394.9 ± 219.49	(2268.1; 2521.6)	9.17
VO_2_ [mL/kg/min]	Max	55.4 ± 2.92	(53.7; 57.1)	5.27
	AnT	42.5 ± 1.49	(41.7; 43.4)	3.50
	AeT	31.5 ± 0.95	(30.9; 32.0)	3.02
VO_2max_ [%]	AnT	76.9 ± 1.73	(75.9; 77.9)	2.25
	AeT	56.9 ± 1.73	(55.9; 57.9)	3.04
VE [L/min]	Max	156.1 ± 19.90	(144.6; 167.6)	12.75
	AnT	112.7 ± 15.03	(104.0; 121.4)	13.34
	AeT	50.5 ± 8.93	(45.4; 55.7)	17.68
RF [b/min]	Max	63.2 ± 5.04	(60.3; 66.1)	7.98
	AnT	47.2 ± 4.73	(44.4; 49.9)	10.03
	AeT	32.7 ± 3.48	(30.7; 34.7)	10.65
HR [bpm]	Max	194.9 ± 4.51	(192.3; 197.5)	2.31
	AnT	171.3 ± 7.56	(166.9; 175.7)	4.41
	AeT	124.4 ± 5.85	(121.1; 127.8)	4.70
V [km/h]	Max	22.1 ± 1.43	(21.3; 23.0)	6.46
	AnT	18.4 ± 2.70	(16.8; 20.0)	14.68
	AeT	12.9 ± 1.79	(11.9; 14.0)	13.84
VO_2max_ Est. [mL/kg/min]	Max	55.7 ± 3.00	(54.0; 57.5)	5.38
La 30–15_IIT_ [mmol/L]	Max	9.4 ± 1.26	(8.7; 10.1)	13.40
∆La 30–15_IIT_ [mmol/L]	-	8.2 ± 1.25	(7.5; 8.9)	15.24

M—Mean; SD—Standard Deviation; CI—Confidence Interval; CV—Coefficient of Variation; VO_2max_—Maximal oxygen uptake; VE—Minute ventilation; HR—Heart rate; RF—Respiratory frequency; V—Velocity; Est.—Estimated; AeT—First ventilatory threshold (aerobic threshold); AnT—Second ventilatory threshold (anaerobic threshold); La—Blood lactate concentration; ΔLa—Lactate concentration increase (maximal value minus baseline value).

**Table 2:** Descriptive statistics of work time and blood lactate concentration parameters obtained during the Repeated High-Intensity Effort (RHIE) on-ice.

Parameter	Set	M ± SD	±95% CI of M	CV
T_TOT_ [s]	-	122.8 ± 12.21	(115.7; 129.8)	9.94
T_S#_ [s]	S1	41.2 ± 4.02	(38.9; 43.6)	9.75
	S2	40.8 ± 4.05	(38.5; 43.1)	9.93
	S3	40.8 ± 4.25	(38.3; 43.2)	10.42
La_Max_ RHIE [mmol/L]	-	10.0 ± 1.62	(9.1; 10.9)	16.20
La R15 RHIE [mmol/L]	-	4.7 ± 1.95	(3.6; 5.8)	41.76
∆La RHIE [mmol/L]	-	8.7 ± 1.59	(7.8; 9.6)	18.32

M—Mean; SD—Standard Deviation; CI—Confidence Interval; CV—Coefficient of Variation; T_TOT_—Total work time across all series; T_S1_, T_S2_, T_S3_—Total work time in set 1, 2, and 3; La_max_—Maximal blood lactate concentration; La R15—Value at the 15th minute of recovery (restitution); ΔLa—Lactate concentration increase (maximal value minus baseline value).

**Table 3:** Energy system contributions during the Repeated High-Intensity Effort (RHIE) on-ice.

Parameter	Component	M ± SD	±95% CI of M	CV
W_TOT_ [kJ]	-	562.0 ± 52.00	(532.0; 592.0)	9.25
W_TOT_ [kJ/kg BM]	-	7.4 ± 0.53	(7.1; 7.7)	7.16
W [kJ]	AER	352.8 ± 33.84	(333.3; 372.4)	9.59
	BLC	41.2 ± 8.44	(36.3; 46.1)	20.48
	PCr	167.9 ± 24.86	(153.6; 182.3)	14.80
W [kJ/kg BM]	AER	4.6 ± 0.31	(4.5; 4.8)	6.68
	BLC	0.5 ± 0.10	(0.5; 0.6)	18.52
	PCr	2.2 ± 0.34	(2.0; 2.4)	15.32
W [%]	AER	63.1 ± 2.55	(61.6; 64.6)	4.04
	BLC	7.4 ± 1.55	(6.5; 8.3)	20.92
	PCr	29.8 ± 2.91	(28.1; 31.5)	9.77

M—Mean; SD—Standard Deviation; CI—Confidence Interval; CV—Coefficient of Variation.

## Data Availability

The raw data supporting the conclusions of this article will be made available by the authors on request.

## Abbreviations

The following abbreviations are used in this manuscript:

Max	Maximal
AnT	Anaerobic Threshold
AeT	Aerobic Threshold
W_AER_	Aerobic Component
W_BLC_	Anaerobic Lactic (Glycolytic) Component
W_PCr_	Anaerobic Alactic (Phosphagen) Component
VO_2max_	Maximal Oxygen Uptake
VE	Minute Ventilation
RF	Respiratory Frequency
HR	Heart Rate
V	Velocity
La	Blood Lactate Concentration
W	Work (Energy Expenditure)
W_TOT_	Total Energy Expenditure
30–15_IIT_	30–15 Intermittent Ice Test
RAT	Repeated Ability Test
RHIE	Repeated High-Intensity Effort
